# Unlocking policy synergies, challenges and contradictions influencing implementation of the Comprehensive Sexuality Education Framework in Zambia: a policy analysis

**DOI:** 10.1186/s12961-023-01037-y

**Published:** 2023-09-14

**Authors:** Malizgani Paul Chavula, Joseph Mumba Zulu, Isabel Goicolea, Anna-Karin Hurtig

**Affiliations:** 1 Department of Epidemiology and Global Health, Umeå University, 901 87 Umeå, Sweden; 2 Department of Health Policy Management, Promotion and Education, School of Public Health, The University of Zambia, Ridgeway Campus, Box 50110, Lusaka, Zambia

**Keywords:** Comprehensive sexuality education, Sexual reproductive health rights, Policy analysis, Facilitators, Challenges, Adolescents

## Abstract

**Background:**

Comprehensive sexuality education (CSE) has recently become salient, but adolescent sexual reproductive health and rights (ASRHR) challenges are still a global health problem. Studying policies which have implications for CSE implementation is a crucial but neglected issue, especially in low and middle-income countries (LMICs) like Zambia. We analyzed policy synergies, challenges and contradictions influencing implementation of CSE framework in Zambia.

**Methods:**

We conducted a document review and qualitative interviews with key stakeholders from Non-Governmental Organizations, as well as health and education ministries at the National and all (10) provincial headquarters. Our methods allowed us to capture valuable insights into the synergies, challenges and contradictions that exist in promoting CSE framework in Zambia.

**Results:**

The study highlighted the synergies between policies that create opportunities for implementation of CSE through the policy window for adoption of sexual reproductive health and rights (SRHR) that opened around the 1990s in Zambia, promotion of inclusive development via education, adoption of an integrated approach in dealing with SRHR problems, and criminalization of gender-based violence (GBV). This analysis also identified the policy challenges and contradictions including restricted delivery of education on contraception in schools; defining childhood: dual legal controversies and implications for children, grey zones on the minimum age to access SRHR services; inadequate disability inclusiveness in SRHR legal frameworks; policy silences/contentious topics: LGBTQI + rights, abortion, and grey zones on the minimum age to access SRHR services.

**Conclusion:**

While many policies support the implementation of CSE in schools, the existence of policy silences and challenges are among the barriers affecting CSE implementation. Thus, policy reformulation is required to address policy silences and challenges to enhance effective promotion and integration of the CSE framework.

## Background

Many low and middle-income countries (LMICs) are integrating comprehensive sexuality education (CSE) to promote adolescents’ sexual, reproductive health and rights (ASRHR), in line with Agenda 2030 on acceleration of the attainment of universal health coverage for all [1–4]. According to UNESCO (2017: 16–17) Comprehensive sexuality education is defined as:

“*the curriculum-based process of teaching and learning about the cognitive, emotional, physical, and social aspects of sexuality. It aims to equip children and young people with knowledge, skills, attitudes, and values that will empower them to: realize their health, wellbeing, and dignity; develop respectful social and sexual relationships; consider how their choices affect their wellbeing and that of others; and understand and ensure the protection of their rights throughout their lives.”*[5]

The promotion of ASRHR is also crucial in creating a healthy socio-economic development environment and wellbeing [4]. Comprehensive sexuality education has recently become salient, but adolescent SRHR challenges are still a global health problem. Socio-economic and cultural factors and gender norms place adolescents and the youth, especially girls, at higher risk than other age groups of sexually transmitted infections (STIs), including HIV [6, 7]. Studies have also estimated that 10 million child marriages occur every year [7].

Promoting ASRHR goes beyond providing CSE, it requires interventions that reduce structural inequalities, challenge norms, legal reforms, strengthening health systems and social economic development. However, this does not mean that CSE is not important; the educational system is a vital setting for health promotion and education for adolescents’ development through CSE. There are numerous health and wellbeing benefits of implementing CSE within the education system. CSE provides adolescents and young people with foundational SRHR knowledge and competencies during their formative years [8]. CSE has been associated with great potential to provide adolescent, and the youth with essential information and skills about how their body functions, on sexuality, and to reduce misinformation, shame, and anxiety, and to improve their abilities to make safe and informed choices about their sexual and reproductive health [9, 10].

The global evidence on delivering CSE shows that it has also been associated with improved health outcomes, including reduction in adolescent pregnancies, unsafe abortion, sexual and gender-based violence, and promotion of gender equality and adolescent’s people’s overall SRHR [4, 9–14]. Other studies also suggest that good quality implementation of CSE has been associated with positive effects on sexual knowledge, attitudes, communication skills, and certain sexual behaviours [10]. Moreover, CSE may also support adolescent, especially girls, in gaining knowledge about puberty, menstruation, and promoting a safe passage to adulthood and in reaching their full potential in educational achievement, earning capacity, and societal participation [10]. This also contributes to skills’ development, employability, and empowerment opportunities for adolescents that are essential for socio-economic development of countries [10].

Despite these proven CSE benefits, some stakeholders such as community, religious leaders, and parents oppose the teaching of CSE because it is believed to deliver content areas they perceive to be in conflict with dominant religions and cultural practices, for example on lesbian, gay, bisexual, transgender, Queer, Intersex, and plus (LGBTQI +) rights, and contraceptive use [15, 16]. In addition, the legal environment in some countries including West and East Africa limit adolescents’ access to sexual, reproductive health services and education [13, 17–21]. These contextual factors and the policy landscape have shaped the implementation of the CSE framework in many LMICs. However, to date there is limited research attention on policy synergies, challenges and contradictions influencing the implementation of the Comprehensive Sexuality Education Framework in Zambia.

## Comprehensive Sexuality Education Framework in Zambia

Promoting ASRHR, especially in relation to preventing challenges such as early and unwanted pregnancy and marriage, is on the socio-economic and political agenda in Zambia. The government has introduced policies aimed at reducing challenges related to SRHR, including the prevention of adolescent pregnancies and marriages, while promoting the overall adolescent well-being and health. Some of the ASRHR related policies include the Re-Entry Policy 1997, The Education Act of 2011, the Adolescent Health Strategy, and National AIDS Council. The introduction of SRHR related policies contributed to the development of CSE content in Zambia. The Reproductive Health Education Act (law) of the 1990s was replaced by a new Comprehensive Sexuality Education Framework (2014). The new CSE framework added more SRHR themes such as gender relations, information on contraceptive methods, sexuality, as well as values, attitudes, and self-realization life skills.^4^ The new framework was revised in line with UNESCO’s international guidelines on sexuality education (2009). The Ministry of Education in 2014 set out the new and ambitious framework for CSE [20], which is summarized in Table 1. The Comprehensive Sexuality Education Framework was integrated into career subjects and introduced in all schools in Zambia. The framework targets children, and adolescents enrolled in grades 5–12 (about 10 to 19 years old) in schools as a way of addressing the health knowledge gap [20]. The overarching purpose of implementing CSE in Zambia has been to address the inadequate and unequal access to sexual and reproductive health (SRH) knowledge among young people, which contributes (among other factors) to several SRH-related problems [20]. The programme has created opportunities for adolescents to acquire life skills and knowledge.

**Table 1:** Outline of thematic areas included within the Comprehensive Sexuality Education Framework

First thematic areas	Second thematic areas
1. Relationships	4. Human Development
1.1 Families	4.1 Sexual and Reproductive Anatomy and Physiology
1.2 Friendship, Love, and Relationships	4.2 Reproduction
1.3 Tolerance and Respect	4.3 Puberty
1.4 Long-term Commitments, Marriage, and Parenting	4.4 Body Image
2. Values, Attitudes, and Skills	4.5 Privacy and Bodily Integrity
2.1 Values, Attitudes, and Sources of Sexual Learning	5. Sexual Behaviour
2.2 Norms and Peer Influence on Sexual Behaviour	5.1 Sex, Sexuality, and the Sexual Life Cycle
2.3 Decision-making	5.2 Sexual Behaviours and Sexual Response
2.4 Communication, Refusal, and Negotiation Skills	6. Sexual and Reproductive Health
2.5 Finding Help and Support	6.1 Pregnancy Prevention
3. Culture, Society, and Human Rights	6.2 Understanding, Recognizing, and Reducing the Risk of STIs, including HIV
3.1 Sexuality, Culture, and Law	6.3 HIV and AIDS Stigma, Treatment, Care, and Support
3.2 Sexuality and the Media	
3.3 The Social Construction of Gender	
3.4 Gender-Based Violence (GBV), Sexual Abuse and Harmful Practices	

Curriculum Development Centre (2013), Comprehensive Sexuality Education Framework

The CSE programme has been implemented in partnership with UN agencies such as UNESCO, UNFPA, UNAIDS, and other local organizations. However, the development and dissemination process of the CSE content left out many key stakeholders including religious leaders, civic leaders, parents, youth, and civil society organizations.^2, 3^ The implementation process has been characterized by contestation and opposition due to the perceived incompatibility of CSE with cultural and religious values [20].

Studies on CSE conducted in Zambia have focused on documenting the implementation process of CSE in youth clubs in relation to barriers and facilitators affecting implementation and acceptability of the programme in externally funded projects [19, 20, 22, 23]. Health policies and laws are crucial in providing guidance towards implementation of complex interventions, however policy domains may clash thereby negatively influencing the implementation of interventions [24]. It is also rare to find any study that explicitly explores the policy domains that support or hinder implementation of CSE in Zambia or similar contexts. The limited research attention on the policy landscape influencing the implementation of the Comprehensive Sexuality Education Framework in Zambia remains a significant grey area. Without a thorough documentation on how the policy environment influences the implementation of CSE, opportunities may be missed to develop effective strategies to overcome challenges that hinder optimal success. Therefore, this study aims to fill this knowledge gap by analysing policies with the view of identifying synergies challenges, and contradictions influencing implementation of comprehensive sexuality education in Zambia.

## Methodology

### Study context and site

Zambia is a low middle-income landlocked country, located in Southern-Central Africa. Low economic and social development are among the major drivers of poverty in Zambia. Zambia has been ranked by the World Bank as one of the countries with highest poverty levels with over 61% out of 19.6 million population earn less than the global poverty line of $2.15 per day [25, 26]. This heightened vulnerability exacerbates pathways to various SRHR concerns experienced by adolescent and young people. The Zambia Demographic and Health Survey (2018–2019) reported that adolescents and young people experience several SRHR challenges. For example, 29% of adolescent girls get pregnant before the age of 19 and 15% get married [27]. This prevalence is higher in Southern, Eastern and Central provinces compared to other provinces in Zambia. Furthermore, about 25% of married girls aged 15–19 have an unmet need for family planning [9]. The burden of HIV among adolescent and young people is 3.8%, with young women being more affected: about 6% compared to young men at about 2% [27]. While CSE was already launched in Zambia in 2014, studies report that adolescents still have limited access to school-based sexuality education [28], and that the implementation process has been complex [29]. In addition, cultural barriers hinder adolescents from discussing sexual and reproductive health concerns with parents [28].

### Study design and data collection methods

This study adopted a qualitative policy analysis design which involved policy review and full participation of key stakeholders from ministries of health, and education, and their partners from Non-Governmental Organisations (NGOs). This study design was selected to facilitate collection of in-depth contextual information on policy synergies, challenges and contradictions influencing implementation of the Comprehensive Sexuality Education Framework in Zambia. This study consisted of a review of policy documents, and qualitative interviews to explore policy opportunities and challenges influencing implementation of comprehensive sexuality education in Zambia. The policy review was useful to understand how policy content domains affect implementation of the programme (CSE framework). We also conducted 49 qualitative key informant interviews (KII) to understand key stakeholders’ perspectives regarding key policy issues that influence implementation of CSE policy in Zambia. In conducting this study, we employed the consolidated criteria for reporting qualitative research (COREQ) when preparing this manuscript [30]. The adopting of the COREQ checklist enabled us to comprehensively report essential aspects of the research such as study design, and analysis and findings, thereby enhancing transparency and rigor of this study.

### Policy analysis

A document review approach was employed to analyze existing policies influencing CSE implementation. The analysis identified policy content domains that either support or hinder CSE policy implementation in Zambia. To find relevant documents appropriate for policy analysis, the following criterion was used: a policy or programme should be relevant in influencing implementation of ASRHR services. These policies may be pre-existing, a modification of existing policy, or new policy in response to addressing SRHR challenges. The policy may apply to any sector promoting adolescent SRHR. The reviewed documents included the laws (Acts of parliament) and government policy documents on SRHR related matters.

We purposefully searched for SRHR related documents from government department (including education, health, gender, justice) websites. We further reached out to various government departments to collect relevant documents that guide SRHR implementation. Additional documents were also sourced through searches of references in selected documents. The entire search yielded a total of 12 documents that were eligible for policy analysis. The data extracted provided information on policy content domains supporting and hindering CSE implementation are shown in (Table 2) below.

**Table 2:** Policies reviewed for this study

Authors/Policy Sector Origins	Year	Policy Title	Policy Issues Relevant to the Study
Ministry of Education	1996	Educating Our Future	This policy guides implementation of education in Zambia, highlights how social and economic challenges affect progression of pupils and integration of SRHR related strategies into the educational systems.
Ministry of Education	1997	Re-Entry Policy	This policy stresses the significance of re-engaging girls who have dropped out of school due to pregnancy and parenting to return to the education system.
Ministry of Education	2011	Education Act	The policy promotes education by prohibiting marriages among learners and supports integration of SRHR related education into the school system
Ministry of Education	2014	Comprehensive Sexuality Education	The framework acts as a guide on how schools can integrate SRHR, GBV, HIV, respect, values, and life skills into various key career subjects.
Ministry of Health	2017-2021	Adolescent Health Strategy	The policy focuses on strengthening the delivery of adolescent responsive health services, to increase adolescent access and utilization of quality health care leading to improved SRH and the reduction of HIVAIDS, and promotion of healthy living among adolescents. Scales up implementation of comprehensive sexuality education.
Ministry of Health	2022	HIV Counselling Testing Guidelines	The policy provides for age of consent at which a person can access HIV services on their own.
Ministry of Gender	2011	Anti-Gender Based Violence (GBV) Act	The policy highlights various GBV cases including sexual violence, child marriage, and abuse.
Ministry of Justice	1918	Marriage Act	Law exempts all marriages under any African customary law from the minimum age of marriage requirements (normally 21 years) under the law.
Sections 17 and 34 of Zambia’s Marriage Act		Customary Law	The law does not provide a minimum age of consent to marry under Zambian customary law as current customary practice allows any girl who has attained puberty to get married.
Ministry of Legal Affairs, Government of the Republic of Zambia	2012	Penal Code Act	The Act guides on how to handle crimes on GBV, sexual violence, child abuse (child pregnancy and marriages). Any person who commits the offence of rape is liable to imprisonment for life. The act criminalizes same sex sexual relationships and marriages.
Ministry of Legal Affairs, Government of the Republic of Zambia	1972/1994	The Termination of Pregnancy Act	Decriminalizes abortion in cases when the pregnancy would have involved a risk to the life of the pregnant woman or child greater than if the pregnancy were terminated
Ministry of Justice	2021	*Zambia Law and Development Commission*	A girl is considered capable of marrying at puberty although some ethnic groups allow a longer period for a girl to be more mature. On the other hand, a boy is considered ready for marriage once he grows a beard and shows an ability to do work that can support a wife, children and other members of the family. Customary marriages are potentially polygamous [4].

### Qualitative interviews

In addition to collecting secondary information, a primary data collection which consisted of key informant interviews was conducted from January 2021 to April 2021. This study is part of the bigger PhD project “the role of collaborative governance in influencing implementation of comprehensive sexuality education framework in Zambia” where a total of 106 key informant and interviews were collected. However, 49 key informant interviews were selected and analyzed, as data saturation was reached under this study. We conducted key informant interviews with key stakeholders representing Ministry of Education and Health, and NGOs at national and all provincial districts in Zambia (Table 3). We employed a purposive sampling strategy to select interviewees from relevant mentioned sectors. The national coordinators of CSE and SRH from the Ministry of Education and Health helped us to identify a list of key informants. This approach enabled us to deliberately select participants who held significant expertise in relation to their involvement in implementation and coordination of CSE framework. The interview guides explored the policy synergies, challenges, and contradictions in relation to policies and guidelines that support or hinder implementation of CSE. All interviews were conducted in the English language by the first author (MPC) with the help of trained research assistants. The first author has a Master of Public Health qualification and vast experience in conducting qualitative research. The research assistants had bachelor’s and master’s degrees in public health and social sciences. All the interviews were recorded using audio tape recorders, and then transcribed verbatim. Each individual interview lasted around 30–90 min.

**Table 3:** Key informant interviews

Sector	Key informant interviews	Conducted KIIs
Ministry of Health	National level (policy maker)	1
	Provincial level	10
	District level	8
	Subtotal	19
Ministry of Education	National level (policy maker)	1
	Provincial level	9
	District level	10
	Subtotal	20
Non-Governmental Organization	Involved in policy implementation	10
Grand Total		49

### Data analysis

This study adopted the qualitative content analysis approach by Graneheim and Lundman to analyze the data [31]. This approach enabled us to systematically describe a phenomenon, highlighting differences and similarities in terms of patterns and communication processes [31]. The transcribed interviews and policy documents on CSE were imported into NVivo (QSR International UK, 2021) to support data management and analysis. Each transcript and policy document were considered as a unit of analysis for this study. The interviews and policy documents were read through many times to understand policy content domains that influence implementation of CSE in Zambia. The appropriate codes from each meaning unit were developed and written. Using the latent content analysis to uncover the underlying meaning and condensed meaning units into categories. This process involved closely exploring the text, condensing the manifest content into a meaning description, and interpreting the latent content to examine deeper meaning. Through this approach, developed categories were identified and analyzed to gain a full understanding of policy issues that influence implementation of CSE framework in Zambia. The codes were sorted into two *content domains* reflecting policy synergies*,* challenges, and contradictions to the implementation of the CSE framework. Within each of these content domains, provisional categories were developed through sorting the codes into groups. The provisional categories were discussed and revised among the four authors (MPC, JMZ, IG, AKH) until the final categories for each content domains were developed (as in shown Table 4 below).

**Table 4:** Major content domains and categories – synergies, challenges and contradictions influencing implementation of CSE

Major content domains	Categories
Policy synergies for CSE implementation	a. Policy window for adoption of SRHR opened around the 1990s in Zambia. b. Promotion of inclusive development via education c. Adoption of an integrated approach in dealing with SRHR problems. d. Strategies that aim to prevent adolescent pregnancies and child marriages. e. Criminalization of GBV
Policy challenges, contradictions, and silences	a. Restrictive delivery of contraceptives in schools b. Defining childhood: legal controversies and implications for child marriage c. Grey zones on age of sexual consent and access to SRH services d. Inadequate disability inclusiveness in SRHR legal frameworks e. Policy silences / contentious topics: LGBTQI + rights and abortion

## Results

### Policy domains of synergies, challenges-contradictions, and silences

Policy synergies (opportunities) refer to particular domains within policies that facilitate or support the effective implementation of the CSE framework within the school system in Zambia. Whereas policy challenges and contradictions included various policy domains or factors and issues that hinder the successful implementation of the CSE framework in the school system. The content analysis categories were developed within two main content domains: synergies, challenges-contradictions, and silences influencing CSE implementation. Policy implementation opportunities included: policy window for adoption of SRHR that opened around the 1990s in Zambia, promotion of inclusive development via education, adoption of an integrated approach in dealing with SRHR problems, strategies that aim to prevent adolescent pregnancies and child marriages, and for the criminalization of GBV. The policy challenges and contradictions including restrictive delivery of contraceptives in schools; defining childhood: dual legal controversies and implications for children, grey zones on the minimum age to access SRHR services; inadequate disability inclusiveness in SRHR legal frameworks; policy silences / contentious topics: LGBTQI + rights; abortion; and grey zones on minimum age to access SRHR services (Table 4).

### Policy synergies for CSE implementation

#### Policy window for adoption of SRHR opened around the 1990s in Zambia

The emerging global and national interest towards creating interventions that address SRHR issues was one of the major drivers for recognizing the importance of integrating CSE into the educational system in Zambia:

*“Areas of major national concern include HIV/AIDS…young people frequently experience problems arising from their developing sexuality, and the general health of the people and children of Zambia. It is imperative that the basic school curriculum deal with these issues [population education, sexuality, HIV/AIDS, interpersonal relationships], striving to create attitudes and establish practices that will be conducive for good health and personal wellbeing.” (Educating Our Future, 1996) *[32]

The consequences of the high prevalence of SRH problems including HIV, STIs, pregnancies, and marriages among adolescent and young people getting media and political attention contributed to triggering change in the SRHR policy landscape. The international and local partners and interest groups advocated for the introduction of adolescent friendly sexual and reproductive health services. In Zambia, the policy window was opened around the 1990s which highlighted that educational institutions should adopt strategies that promote sexual and reproductive health and rights for learners. Some of the introduced policies, including Educating Our Future, 1996 [33] and the Re-Entry Policy, 1997, [34] stressed the need for educational systems to develop strategies that promote SRHR. These initial policies in relation to CSE focused on abstinence while later, around 2014, policies became much broader. The 2014 CSE framework, for example, includes much more content on SRH such as gender relations, sexual behaviour, contraceptive methods, values, attitudes, and self-realization life skills. Furthermore, the policy space in Zambia has enhanced the integration of sexual and reproductive health services, for example the use of a peer educator approach to deliver youth friendly SRHR services.

#### Promotion of inclusive development via education

Education has been recognized as a crucial tool for reducing inequalities and social development (Educating Our Future, 1996). Some policies address social determinants that affect access to education. Factors such as poverty, hunger, illiteracy, unemployment, and vulnerability among families and community are some of the issues which have been targeted in the policies as shaping low access to education. The challenges faced by parents such us their inability to provide education support, i.e., school fees and school materials (books, shoes, and bags) to their children. Therefore, the Ministry of Education introduced programmes and policy that aim to address such inequalities to access education among adolescents. Policies including the Education Act and others support education progression by providing scholarships and bursary schemes to students in vulnerable situations, such as orphans, who cannot afford to pay for their education. This is based on the understanding that providing education to all learners, especially girls, also creates opportunities for adolescents to learn about CSE, stay in school longer, and avoid pregnancies. This helps young people to acquire self-efficacy in making sexual decision making.

*“This widespread poverty affects education in many ways. Many of the poor have little understanding of the extensive benefits of education…they may attach little value to school attendance preferring to employ their children in the home, on the farm trading, in petty trading…with their little financial resources, they may not be able to afford the costs arising from the school participation of their children or to buy stationery and learning materials. They must not be denied access through inability to pay school related expenses like uniforms, materials…the ministry will introduce bursary and scholarship for the needy.” *[35]

Some programmes such as Keep Girls in School have introduced strategies for re-engaging girls who drop out of school due to pregnancy back into the education system. To re-engage, they are given educational incentives including bursaries. According to a policy implementer in the Ministry of Education:

*“We have the Keep Girls in School (KGS), program for advancement of girl children that policy also is mobilizing girls who are fallen out of school so that they can come back, then those ones who are still in school we are trying to do the same for them so that they don't follow the routes of their colleagues who at one time left school… dropped out at grade seven, they dropped out at grade nine, in grade twelve” (KII 37, MoE).*

#### Adoption of an integrated approach in dealing with SRHR problems

The adolescents and young people have many different needs when it comes to SRHRs. Thus, the reviewed policies (Educating Our Future, Adolescent Health Strategy, and Education Act) acknowledged the significance for the education sector to introduce strategies that deal with cross-cutting adolescent SRHR issues. These policies outlined how various SRHR issues: STIs, HIV/AIDS, counselling, child protection, gender relations, and life skills, could be integrated into the educational system [32, 33, 35].

*“Integration of sexuality, and relationship, HIV/AIDs and life skills teaching into the school curriculum…the Ministry supports Anti-AIDS programme and Anti-AIDS clubs in schools will continue as these are spearheading an important awareness movement that is gradually reaching out to every pupil.” (Educating Our Future, 1996 *[33]

Furthermore, some policies also stressed the importance of ensuring that CSE is implemented across the country to help adolescents in and /or out of school to have opportunities to increase their awareness of SRHR issues.

*“Design and implement targeted innovative behavioural change campaigns …which promote healthy behavioural development and behaviour change; and preventative health services, including the scaling up of CSE for adolescents in and out of school.” (Adolescent Health Strategy, 2017)*

The participants expressed the support for an integrated approach in delivering CSE. The integration in delivery of CSE entails that all crucial aspects of SRHR including prevention of adolescent marriages and pregnancies, HIV, and gender-based violence, are emphasized in school. This approach provides a chance for learners to acquire necessary skills and topics covered in the CSE framework. The Comprehensive Sexuality Education Framework is perceived as empowering learners with knowledge and skills to help them attain self-efficacy in helping them make better SRHR decisions, including helping girls to prevent pregnancy and delay marriage while they are pursuing their education. The key informant emphasized the key role of offering all crucial SRHR issues to adolescents:

*“A child needs to understand their rights in terms of SRHR, HIV/AIDS, human rights, gender equality, pregnancy “prevention.” (KII 09, Female MoE Staff)*

*“CSE is the type of education, which is inclusive, it touches all areas of life just to ensure that we have a child who is holistically developed in the way they make their decisions especially concerning sex and their health at large.” (KII 36, Female, Staff)*

#### Strategies to prevent adolescent pregnancies and child marriages

Many girls drop out of school due to pregnancies and marriages. Thus, some policies within the educational sector advocate prevention of unwanted adolescent pregnancies and marriages. This space gives authority for SRHR programmes like CSE to be implemented with the aim of contributing to the reduction of SRHR problems.

*“Prevent or stop a learner who is a child from attending school for the purpose of marrying or marrying off the learner who is a child.” (Education Act, 2011)*

Despite the introduction of these polices, unwanted child pregnancies remain a major problem. Some polices including the re-entry policy support the development and implementation of strategies that facilitate engaging girls who dropped out of school back into the education systems. Adolescent pregnancy has been reported to be associated with shame and stigmatization; hence the policy also stresses the provision of counselling and the social system to encourage girls to continue with their education. In addition, parents, teachers, and the community should also be engaged in the provision of emotional and social support during maternity leave. This is crucial to avoid further occurrence of unwanted pregnancies and getting married.

Participants, however, perceived the re-entry policy and similar initiatives as a double-edged sword that encourages adolescent pregnancy since the pupils are given opportunities to return to school. From their perspective, this appeared to contradict the lessons in CSE and other interventions that stressed the importance of prevention of adolescent pregnancy and marriages among learners.

*“Re-entry Policy is a provision where children who have fallen pregnant before can be allowed back to school to continue, but we are saying even that delay of going for maternity leave is not good. That’s why we are saying implementation of CSE tends to even overlook the re-entry point… once you start having sex it is difficult to stop completely that’s why in our counselling service for the re-entered girls we give the options of contraceptives, much as we don’t encourage them.” (KII 37, MoE Staff).*

#### Criminalization of GBV

Gender-based violence is described as a major problem, and policies expressed the significance of protecting society, especially girls, against GBV. The laws describe different forms of GBV crimes and establish different penalties associated with them. This may include life imprisonment for perpetrators of GBV in some cases. The expectation is that perpetrators may refrain from committing these crimes for fear of the strong penalties associated with them, and the community may be more prone to report such cases to relevant authorities.

*“Any person who unlawfully and carnally knows (or sexual harassment, cleansing, exploitation, female genital mutilation) any girl under the age of sixteen years, is guilty of a felony and is liable to imprisonment for life or 14 years…” (Penal Code, 2012)*

Collaboration among stakeholders is key to obtain their support and commitment towards prevention and management of GBV. The school management and teachers can work together with other sectors in the prevention of GBV, and this gives an opportunity for stakeholders to collectively implement of the programme.

*“In cooperation with the Ministry responsible for education, offer a programme aimed at the provision of education to child victims.” (Anti Gender Based Violence Act, 2011)*

*“Achieve cultural and value shifts through changes in social norms and behaviour, like GBV, child marriage, alcohol, and substance abuse.” (Adolescent Health Strategy, 2017)*

### Policy challenges; contradictions, and silences

Policy challenges were those content domains influencing implementation of CSE in schools. We identified four categories: restrictive delivery of contraceptives in schools; defining childhood: dual legal controversies and implications for children, grey zones on the minimum age to access SRHR services; inadequate disability inclusiveness in SRHR legal frameworks; policy silences / contentious topics: LGBTQI + rights, and abortion, and grey zones on minimum age to access SRHR services.

#### Restrictive delivery of contraceptive in schools

Some policies in the health sector have stressed the significance of delivery of adolescent friendly and responsive SRHR services including contraceptives in schools, health facilities, and communities among other places [29]. The school systems have been seen as a crucial agency to create demand for access to SRHR services including contraceptives. Some policies reported the importance of delivery of SRHR services including condoms in school settings to help young people to use them and prevent unwanted pregnancies and sexually transmitted infections [29]. Similarly, education sector policies acknowledge the importance of creating strategies such as education on SRHR, but distribution of any contraceptives, including condoms, is not allowed in school settings [32, 33].

*“Distribution of condoms in the schools they are not comfortable with that. They just refer them (pupils) to the health facilities to get condoms... in the schools we teach these pupils to refrain from activities here.” (KII 65, MoH Staff)*

*“CSE… empowers pupils with knowledge on abstinence, culture & society, hygiene, human development, family planning in prevention of pregnancy, and HIV, life skills and values to teenagers to be more assertive and careful with their sexuality.” (01, Male NGO Staff)*

“*We provide sexual reproductive services for young people, so when they taught in school maybe on family planning is one of the topics they’re taught, and remember Ministry of Education, (distribution of) condoms… Is not even allowed in schools, they…access them if they want to use them, they must come to the facility, so those linkages have been taking place.”* (17, Female, Staff, Provincial Coordinator, MoH)

School systems and health facility collaboration is essential in promoting access and uptake of SRHR services. This creates an opportunity to invite health care providers, community health workers (CHWs), and peer educators to talk about SRHR topics which teachers may feel uncomfortable to discuss with pupils in class. The collaboration, when it works, may contribute to comprehensive discussion on SRHR, and improve the integration of CSE in schools. However, such collaboration has been received with mixed feelings because the health sector has been perceived to be advocating for comprehensive delivery of SRHR services. Thus, the school administration may not want their school promoting the use of condoms that might be against their religious beliefs.

Furthermore, this review also shows the significance of stakeholder collaboration in enhancing the delivery of the SRHR service beyond health information. School, community, and healthcare collaboration may help to facilitate and strengthen referral systems by linking adolescents to health facilities to obtain more SRHR services that cannot be accessed within the school environment.

*“Platform for engaging and advising key stakeholders and leaders on key adolescent health …teenage pregnancies and child marriages. Provide basic health information (ASRR and HIV, etc.), distributes IEC materials and condoms and provides guided referrals to health services in the health centres…” (Adolescent Health Strategy, 2017) *[29]

#### Defining childhood: dual legal controversies and implications for child marriages

The constitution is the supreme law of the land, it defines a child as someone below the age of eighteen years. One policy document defined a child as: *“child means a person who has attained, or is below, the age of eighteen years” (*Constitution of Zambia, 2016). However, the same constitution acknowledges the existence and establishment of other laws on child development and protections. The constitution appears to support culture and traditions though they may compromise child protection rights.

*“The institution of chieftaincy and traditional institutions are guaranteed and shall exist in accordance with the culture, customs, and traditions of the people to whom they apply.”* (Constitution of Zambia, 2016)

Through our analysis, we also identified another definition of the child from the perspective of the Penal Code (Law) which defines a child as someone below the age of 16 years. The 16 years bracket is also the age for sexual consent. This is reflected in the following quote from one of the policy documents: “*Any person who unlawfully and carnally knows any girl under the age of sixteen years is guilty of a felony and is liable to imprisonment for life.”* (Penal Code, 2012). The current legal framework, although defining an age limit, falls short in effectively combating instances of child abuse that occur with victims aged 16 years and above.

Currently, the legal environment permits adolescent marriages at 16 years and above, while any marriages below 16 years are invalid. For instance, in Zambia “*a marriage between persons either of whom is under the age of sixteen years shall be void” (*Marriage Act, 1918). Though this law also promotes adolescent marriages of 16 years and above. However, we also noted that this cannot prevent child marriages of children below 16 years because Zambia recognizes the existence of customary law which allows child marriages below 16 years as further highlighted below.

Our analysis further identified another definition of childhood from Customary Law. For example, Customary Law defines a child as someone who has not attained puberty, regardless of age. Moreover, Customary Law allows anyone to marry at any age as long they have attained puberty and are “physically mature”, and parents or guardians have consented. As one of the policy documents explained:

*“Under customary marriage there is no specific age for marriage.” (Zambia Law and Development Commission, 2021).*

Qualitative findings also reported the complex challenges in defining a child within cultures where adolescent marriages are normal, leading to legal controversies that hinder child protection measures. During the key informant interview, the stakeholder highlighted the legal contradictions in Zambia, saying:

*“There are those contradictions… the statutory law is not clear marriages above 16 years and then we have statutory marriages and so on… Because we have customary law which allows a child to get married with consent from parents.” (KII 67, Female Staff, NGO)*

As noted above, the policy controversies in Zambia regarding child protection policies are because of conflicting definitions of a child in different laws and customs. The inconsistency in age limits and the policy environment can contribute to misinterpretation of the laws due to confusing definitions. This has a huge potential hindering effort to prevent child marriages and abuse, including rape. Moreover, the perpetrators of sexual abuse can exploit these discrepancies to evade justice, and confusion around the definition of child marriages and abuse may limit the comprehensive delivery of CSE topics around child protection and gender relations aspects in school systems and communities.

#### Grey zones on minimum age to access SRHR services

Zambia’s HIV counselling and testing guidelines align with WHO recommendations, setting the minimum age at 16 years. However, the Penal Code law does not offer explicit guidance on the age of sexual consent in relation to delivery of SRHR services to adolescents, leading to controversies and granting healthcare providers discretionary power in delivering comprehensive sexual and reproductive health services to adolescents. Healthcare providers may limit the delivery of SRHR services to adolescents due to the absence of clear guidelines. Allowing adolescents to access SRHR services with the assent of parents and guardians contradicts the principle of confidentiality, and some caregivers with strong religious and cultural values may not allow their children to access SRHR services. The quote from the key informant highlighted the need for clear age guidelines for access to SRH services.

*“Some of it is the age of consent which I think have been indabas [discussion] of late to see as to age appropriate for consent because you find girls who are below age of 15, they are already sexually active and the consent is an adolescent walks in maybe then wants family planning methods, do you give it or do you have to involve the parents so still something.” (16, Female, MoH Staff)*

*“Review/Strengthen the policy… for adolescent health including clear policies and guidelines on age of consent and access to key SRH and HIV services.” (Adolescent Health Strategy, 2017)*

#### Inadequate disability inclusiveness in SRHR legal frameworks

Lack of disability inclusiveness was perceived as one of the major barriers affecting delivery of CSE to pupils living with disabilities. Participants mentioned putting in place strategies to improve this situation. For example, one participant described:

*“We translated some of the materials into sign language and very soon will also have same materials on various health topics in sign language so that we don’t leave people living with disabilities behind.” (KII26, Female, MoE Staff)*

Policies rarely give guidance on strategies for offering CSE to children living with disabilities in Zambia. Persons living with disabilities including pupils and teachers may have different kinds of needs. Therefore, there is a need to package SRHR information in ways that reaches all students, independently of their disability status.

#### Policy silences/contentious topics: LGBTQI + rights and abortion

The Zambian Penal Code Act of 2012 highlights the criminalization of sexual acts ‘*contrary to the order of nature’*:

*“Any person who has carnal knowledge of any person against the order of nature or permits a male person to have carnal knowledge of him or her is committing a criminal offense” (Penal Code Act, 2012)*

Based on this statement within the penal code, same-sex relationships are interpreted as being against the law in Zambia. Consequently, LGBTQ + rights are not covered within the CSE framework. Despite this, respondents perceived that CSE promoted LGBTQI + rights, which was reported as one of the major grounds for opposition to the implementation of CSE due to traditional and religious norms.

Abortion, another contentious topic, is not included in the CSE framework. The Termination of Pregnancy Act (1972) in Zambia allows for legal abortion under certain circumstances, such “*as risks to the life or health of the pregnant woman, existing children, or the child to be born”*. However, due to cultural and religious issues, the topic of abortion is not included in the CSE framework despite the government recognizing its public health implications. The absence of this topic from the curriculum creates some knowledge gaps as many adolescents may not have the opportunity to learn about safe abortions in school.

## Discussion

This study explored policies to identify opportunities and challenges influencing the implementation of the Comprehensive Sexuality Education Framework in Zambia. The key findings on policy opportunities included: the policy window for adoption of SRHR opened around the 1990s in Zambia, the country adopted an integrated approach in dealing with SRHR including HIV problems, and strategies that aim to prevent adolescent pregnancies and child marriages. The study also highlighted the policy challenges “contradictions and silences” affecting policy implementation such as the prohibition of delivery of condoms in schools, the age of sexual consent and marriage, controversies on LGBTQI + rights, and abortions. The discussion focuses on highlighting merging key themes including supportive policy synergy environment, policy contradictions, and silences, and implications of the study.

### Supportive policy synergy environment

The findings of this study suggest that the development of and implementation CSE in Zambia’s educational system were largely influenced by global guidelines on SRHR, including comprehensive sexuality education guidelines (UNESCO, UNFPA, UNAIDS), the International Conference on Population, and Development declarations and the Maputo plan of Action on SRHR [3, 36]. This study reports that Zambia had several policies, laws, and guidelines that supported the implementation of various components of the CSE framework, including prevention of HIV/STIs, adolescent marriages and pregnancies, as well as the promotion of life skills. The policy environment in Zambia provides an enables and support integration of CSE into the educational and school system by leveraging existing educational resources, including human, material, and infrastructure, to promote CSE among learners as major beneficiaries. The existing supportive policy environment provided the scope, direction, and empowerment to key actors to implement the CSE framework. The existence of health policy also motivates actors to collaborate in the implementation of CSE related interventions and empowers them to provide social accountability in providing checks and balances regarding the implementation of CSE Programmes. Studies conducted in LMICs countries also highlights similar evidence that factors such as policy content or design, partial integration, and poor coordination are some of the factors that negatively shape optimal policy implementation [37–40]. Therefore, it is important to ensure that policies are well-designed and fully integrated, and coordination among stakeholders is strengthened to ensure successful delivery of the CSE framework.

### Policy contradictions impact on CSE implementation

Our study also shows how policy challenges inhibit the successful and optimal integration of the CSE framework. Implementing the CSE framework in Zambia faces opposition from actors including community, political, traditional, and religious leaders due to policy contradictions [20]. The policy contradictions around child marriages, age of sexual consent, and contraceptive distribution in schools limit the delivery of ASRHR services. The current policy landscape in Zambia does not have a clear age at which an adolescent can get married, which creates a gap and controversy that negatively affects the optimal integration of CSE into the education and school systems. Furthermore, the study also suggests that the policy misinterpretation by implementers including teachers, who may use their own discretion to decide which aspects of CSE to teach or not, are among factors that further exacerbate the contestation, confusion, and politics regarding what aspects of CSE should be taught or not in schools. Contraceptive, including condoms, distribution in schools is not illegal in Zambia but is in practice prohibited. In Southern and Eastern African studies shed light on how teachers viewed learners as children in need of protection/control, not as adolescents and young people with SRHR rights [41–43]. This perspective may contribute to the current opposition towards distributing contraceptives in schools. This study shows the importance of having clearer policy frameworks and coordination to avoid conflicting perspectives and guidelines, and to enhance support for the optimal implementation of CSE in Zambia.

### Policy silences on abortion and LGBTQ + rights

While Zambia has laws that allow for legal abortions under certain conditions, such as when the life or health of the mother is at risk, abortion is still a controversial and stigmatized topic in the country [44]. Although abortion is legal under certain circumstances, this information is not captured in the CSE framework. The absence of the topic of abortion in the CSE framework in Zambia may be attributed to the same social and cultural factors that hinder access to contraceptives for adolescents. Zambia like other African countries is predominantly influenced by strong conservative religious and cultural values regarding abortion. The Asian Asia evidence also reported beliefs and value systems influence attitudes on abortion, with actors perceiving it as a taboo subject. As a result, many teachers and other stakeholders may be hesitant to openly provide comprehensive sexuality education that includes information about abortion, which limits adolescents’ knowledge on this topic [45, 46]. It shows the need for targeted community education and engagement with communities, policymakers, and traditional leaders to address the stigma and misconceptions on abortion and promote increased access to comprehensive SRHR services for all adolescents. Collaboration is crucial in the delivery of CSE by enhancing collective community ownership in strengthening linkages to SRHR care and uptake of services [47–51].

The Zambian Penal code criminalizes same-sex relationships, which limits the rights of the LGBTQ + community [44]. A study conducted in Bangladesh align with the notion that cultural and religious practices can also contribute to the stigmatization and discrimination of the LGBTQI + community [52]. The inclusion of the LGBTQ + rights topic in the CSE curriculum is currently challenging because it will conflict with the legal framework. In addition, it may trigger strong resistance from religious and traditional leaders that could potentially hinder the implementation of the CSE framework altogether. This highlights the difficult choice of advocating for CSE delivery by arguing that LGBTQ + rights are not included in the curriculum when this exclusion maybe perceived to be compromising the rights of the LGBTQI + community.

## Limitations and strengths of the study

This review represents a comprehensive analysis of several policies and their potential impact on the implementation of CSE in Zambia. However publicly available policy documents may not concisely reflect the implementation of these policies in practice. The study also documented evidence for the need for policy alignment, harmonization, and coordination across different sectors to enhance the implementation of CSE. The findings might be useful to inform the development of more effective policies and strategies which may enhance the implementation of CSE in Zambia and other similar contexts. Additionally, the study contributes to the limited knowledge on policy implementation in the field of CSE in Zambia. Furthermore, the inclusion of actors from the Ministries of Health, Education, and NGOs helped to enhance the trustworthiness of findings by analysing policies influencing the implementation of the CSE framework by different sectors. On the other hand, our study focused primarily on the school context and the perspectives of stakeholders within that setting. As a result, the experiences of out-of-school adolescents accessing comprehensive sexuality education were not explicitly considered. Therefore, more studies should be conducted on factors shaping policy implementation in other settings including communities or health facilities. Finally, some policy documents could have been excluded in this analysis; the study prioritized documents selected through desk review and input from key stakeholders in line ministries. Future studies should consider using a snowballing strategy to enhance sample coverage and include possible additional policy documents.

## Conclusion

This study analyzed policy synergies, challenges, and contradictions influencing the implementation of the Comprehensive Sexuality Education Framework in Zambia. Promoting SRHR is critical for achieving sustainable development and improving the well-being of individuals, particularly adolescent girls, and young women. This study found that a favourable policy environment exists to support an integrated approach in implementing comprehensive sexuality education contributing to increased access to contraception, prevention and treatment of sexually transmitted infections, maternal health, and GBV prevention and response, which is key to achieving positive SRHR outcomes.

The study also found that the existing policy environment embodies strategies that aim to prevent adolescent pregnancies and child marriages, strengthen the criminalization of GBV and promote inclusive development through education. Therefore, stakeholders need to leverage this favourable policy environment to promote implementation of CSE in schools. The inconsistent policy environment presents challenges in promotion of CSE, including exclusion of schools as delivery points for adolescent SRHR services, and discrepancies in laws on defining childhood thereby hindering efforts to mitigate child marriages. Additionally, limited disabilities inclusiveness, and inadequate access to knowledge on abortions limit comprehensive delivery of CSE.

Furthermore, the legal controversies surrounding the definition of a child may have serious implications on child protection policies and hinder efforts to reduce child abuse. It is also necessary for parliament and policymakers to reform these discrepancies and ensure that the legal definitions of a child are consistent and in line with international standards and practices. These challenges require urgent attention by revising policy inconsistencies and gaps that limit implementation of CSE. However, this requires the involvement of all stakeholders, including government departments, NGOs, churches, traditional leaders, communities, persons with disabilities, parents, and adolescents who are key to ensure consensus. The inclusive stakeholders’ consultation may also help achieve policy alignment, policy harmonization, and the enforcement of laws that protect adolescents from engaging in cultural practices such as child marriage. This may contribute to successful integration of CSE into the education system in Zambia. However, the policy reform process to address these challenges should be based on available evidence and data to inform policies and programmes that promote ASRHR. Finally, the CSE framework also requires expansion to include contextual issues such as gender relations and norms. Finally, it will also require targeted education and sensitization of stakeholders, including teachers, to promote a positive perception of delivery of SRHR services.

## Data Availability

The study data can be requested from the author. The articles for this review can be made available upon request.

## Abbreviations

AIDS: Acquired immune deficiency syndrome
CSE: Comprehensive Sexuality Education
CHWs: Community Health Workers
SRHR: Sexual Reproductive Health Rights
SDGs: Sustainable Development Goals
LMICs: Low- and middle-income countries
UNICEF: United Nations Children’s Fund
UNESCO: United Nations Educational, Scientific and Cultural Organization
UNFPA: United Nations Population Fund
NGOs: Non-governmental organization
MDGs: Millennium developmental goals
GBV: Gender Based Violence
MoH: Ministry of Health
MoE: Ministry of Education
